# Gasdermin B (GSDMB) in psoriatic patients–a preliminary comprehensive study on human serum, urine and skin

**DOI:** 10.3389/fmolb.2024.1382069

**Published:** 2024-04-17

**Authors:** Julia Nowowiejska, Anna Baran, Anna Pryczynicz, Justyna Magdalena Hermanowicz, Beata Sieklucka, Dariusz Pawlak, Iwona Flisiak

**Affiliations:** ^1^ Department of Dermatology and Venereology, Medical University of Bialystok, Bialystok, Poland; ^2^ Department of General Pathomorphology, Medical University of Bialystok, Bialystok, Poland; ^3^ Department of Pharmacodynamics, Medical University of Bialystok, Bialystok, Poland

**Keywords:** gasdermin B, GSDMB, psoriasis, cell migration, keratinocytes, pyroptosis

## Abstract

Psoriasis is one of the most common skin diseases and a crucial issue to manage in contemporary dermatology. The search for the details of its pathogenesis, markers and treatment is continuously ongoing. Our aim was to investigate the role of gasdermin B (GSDMB) in psoriasis, the second protein from the gasdermin family, involved in cell death and proliferation. GSDMB serum and urinary concentrations have never been studied in psoriatics, neither tissue expression of GSDMB by immunohistochemistry. The study included 60 psoriatic patients and 30 volunteers without dermatoses as controls. The serum and urinary GSDMB were evaluated by ELISA. Tissue expression of GSDMB was analyzed by immunohistochemistry. The serum and absolute urine concentrations of GSDMB were significantly higher in psoriatic patients than controls without skin diseases (*p* = 0.0137, *p* = 0.039 respectively). Urinary GSDMB/creatinine concentration ratio was significantly lower in patients compared to controls (*p* = 0.0241). The expression of GSDMB in the dermis and epidermis was significantly more prevalent in psoriatic plaque compared to the non-lesional skin and healthy skin of controls (*p* = 0.0012, *p* = 0.017, respectively). Serum GSDMB correlated positively with the age of patients (R = 0.41; *p* = 0.001). Our study adds to the current state of knowledge about psoriasis concerning the potential involvement of GSDMB. Possibly it could be engaged in keratinocytes migration, which requires further research. Elevated serum GSDMB and decreased urinary GSDMB/creatinine concentration ratio could potentially be investigated as psoriasis biomarkers. GSDMB could be investigated in the future as a potential therapeutic target.

## 1 Introduction

Among the vast majority of various skin diseases, psoriasis which, although widely studied, still remains a mystery for scientists. Psoriasis occurs with an average frequency of about 3% and there are about 125 million people suffering from this dermatosis worldwide (Armstrong and Read, 2020). Despite numerous studies, its pathogenesis is still not fully elucidated. Surely, it is an interplay between genetic predisposition and the influence of external stimuli which lead to severe immune disturbances in the skin (Hugh and Weinberg, 2018). Nevertheless, nowadays, psoriasis is perceived as much more than just a dermatosis because it has been associated with many accompanying diseases, often resulting in serious health complications (Oliveira Mde et al., 2015). Among several subtypes of psoriasis, the most frequent is the plaque type. It presents with erythematous-infiltrative lesions with superficial scaling (Rendon and Schäkel, 2019). Being one of the most common dermatoses, psoriasis brought the attention of dermatologists, both in the molecular, and clinical fields.

Relatively recently a group of six proteins called gasdermins have been identified. They have been labeled A-F, whereas the first five are most similar to each other (Zou et al., 2021). The hallmark of gasdermins is the presence of two domains: N-terminal and C-terminal, which are bound by a linker region, the latter domain is able to inhibit the first one (Tsuchiya, 2021). This structure confers the ability to cause pores in plasma membranes in cells, after being cleaved by particular enzymes, causing their death which was called pyroptosis (Tsuchiya, 2021). There has been quite a number of studies on the role of gasdermins but still, they are not fully understood.

Gasdermin B (GSDMB) is a protein from this particular family. Its encoding gene is located on chromosome 17q21 (Zou et al., 2021). It can be cleaved by granzyme A, and as for the caspases influence–it remains inconclusive (Panganiban et al., 2018; Hou et al., 2021; Sarrio et al., 2023; Yin et al., 2023). First studies reported on the GSDMB cleavage by caspases 3, 6, and 7, later papers suggested that there are many more enzymes that could cleave GSDMB, whereas finally there were suspicions that the ability to induce pyroptosis by the cleaved GSDMB depends on its isoform (Sarrio et al., 2023). Indeed, GSDMB has several isoforms that may exert different influence (Oltra et al., 2023; Sarrio et al., 2023). GSDMB is different from other gasdermins because of its lipid binding profile, but also its unobvious engagement in pyroptosis (Wang et al., 2023). GSDMB is characterized by the lack of the ability of autoinhibition but being active in its full-length form or as N-terminal domain solely (Kuc-Ciepluch et al., 2021; Ivanov et al., 2023). Its biological role is not clear and its participation in pyroptosis is not fully elucidated, it may be isoform-dependent (Rana et al., 2022; Kong et al., 2023); its role in cell proliferation and migration has been reported (Rana et al., 2022). GSDMB expression has been observed in the epithelium of the respiratory and gastrointestinal tract, liver, kidneys, or lymphocytes (Zou et al., 2021). So far, GSDMB has been reported in several diseases, most documented in asthma, cancers, and inflammatory bowel diseases (Feng et al., 2018; Kuc-Ciepluch et al., 2021; Zou et al., 2021). In all of the mentioned disorders, tissue overexpression of GSDMB is observed, including in several cancers (e.g., breast, cervical, gastrointestinal tract) (Das et al., 2017; Zou et al., 2021; Rana et al., 2022). GSDMB engagement in infections has been studied as well, e.g., its direct bactericidal influence in *Shigella* invasion (Wang et al., 2023). There are also several links between these disorders and psoriasis. Research has shown that psoriatics have 1.70–2.53-fold increased odds of developing Crohn’s disease (CD) and about 1.75-fold increased odds of developing ulcerative colitis (UC) compared to controls (Fu et al., 2018). They have a common genetic background which involves chromosomal locus 6p21 and IL23R and IL12B genes (Fu et al., 2018), (the prominent involvement of Th17 lymphocytes), dysregulation of microbiota plays a role in both diseases as the reduction in the population of beneficial bacteria occurs (Cottone et al., 2019). Surprisingly, it has been reported that psoriasis is associated with another disease related to GSDMB–asthma (Östling et al., 2019). Apparently, both diseases have similar immune profile, focused on IL17, and patients suffering from asthma in whom high levels of IL17 are detected tend to have more frequent exacerbations, airway neutrophilia or less diverse lung microbiota (Östling et al., 2019). Additionally, there have been several genes discovered that differentially expressed in subjects with high IL17, were the same which are known to be altered in psoriasis (Östling et al., 2019). In this study, we decided to investigate the role of GSDMB in psoriasis. Based on several rationales, we believe it could play a role in its pathogenesis. GSDMB has been suggested to be involved in the proliferation and migration of cells and impaired keratinocytes proliferation and differentiation is a hallmark of psoriasis (Lanna et al., 2019; Rana et al., 2022), moreover GSDMB has been implicated in the pathogenesis of several disorders associated with psoriasis (Das et al., 2017; Cottone et al., 2019; Rana et al., 2022).

In our recent papers, we have proved that GSDMD and GSDME could be involved in the pathogenesis of psoriasis and also potentially become a novel marker of psoriasis (Nowowiejska et al., 2023a; Nowowiejska et al., 2023b). In the current study, we are presenting for the first time the results of combined serum, urine and tissue investigation of GSDMB.

## 2 Materials and methods

The approval for the study was received from the Bioethics Committee of the Medical University of Bialystok (APK.002.303.2022 and APK.002.19.2020) and was conducted by the principles of the Declaration of Helsinki (WMA, 2022). A total of 60 patients (21 women, 39 men) with exacerbation of plaque psoriasis, with a mean age of 50 ± 2.34 years, and 30 gender- and age-matched volunteers without skin diseases and with a negative family history of psoriasis were included in the study on the gasdermin family. Informed consent was signed by all participants before inclusion in the study. Several exclusion criteria from the study were applied: age under 18 years, pregnancy and breastfeeding, other variants of psoriasis than plaque, dietary restrictions, taking oral medications at least 3 months before the study, autoimmune diseases other than psoriasis, renal impairment, and neoplasms. Psoriasis severity was evaluated using the Psoriasis Area and Severity Index (PASI) and was always performed by the same doctor. PASI score is based on the assessment of erythema, infiltration and scale associated with psoriatic skin lesions with regard to the body area involvement. The higher the score, the more severe psoriasis (Žurauskas et al., 2020). The PASI score was considered mild when PASI <10 (PASI I), moderate when PASI 10–20 (PASI II), and severe when PASI> 20 (PASI III). Moreover, basic laboratory tests were conducted before the study began. Serum, urine and tissue samples were obtained from the same cohort and in the manner previously described (Nowowiejska et al., 2023a; Nowowiejska et al., 2023b).

### 2.1 Serum and urine

60 psoriatic patients and 30 sex- and age-matched volunteers without dermatoses have undergone the serum and urine analysis of gasdermin proteins. Laboratory parameters were measured using 8-h fasting blood samples. A total of 9 mL of peripheral venous blood was collected from each participant using vacuum tubes containing the clot activator. They were left at the room temperature to clot for 30 min before centrifugation for 10 min at 2000g to obtain serum. Urine samples were collected as first morning specimens from a mid-stream by aseptic method. No less than 10 mL of urine was collected in a sterile container. Urine samples were centrifugated for 10 min at 2000 g to remove cell debris and the supernatant was aliquoted. The obtained serum and urine were stored at −80°C until further analysis. Basic laboratory parameters were assessed using routine techniques. GSDMB concentrations were evaluated with an enzyme-linked immunosorbent assay (ELISA) provided by EIAab^®^ (Wuhan, China, E15865 h) according to the manufacturer’s instructions by the same investigator in standardized laboratory settings. The minimum detectable dose was 0.312–20 ng/mL. Briefly, 100 µL of standards and test samples (urine, serum) were subjected to a 96-well plate coated with a monoclonal antibody directed against GSDMB, and incubated for 2 h at 37°C.). Then, the liquid was removed and 100 µL of Detection Reagent A was added. The plate was incubated for 1 h at 37°C. After incubation, the plate was rinsed three times using an Elx-50 automated microplate washer (BioTEK^®^, Winooski, Vermont, USA). Next, 100 µL of Detection Reagent B was added to each well, and incubated for 1 h at 37°C.). Subsequently, the plate was washed five times and TMB substrate solution was added to each well, and a colorimetric reaction was observed. After incubation, the enzymatic reaction was terminated, and the concentration of GDSMB was measured spectrophotometrically at 450 nm using a Multiskan FC microplate reader (ThermoScientific^®^, Waltham, USA). Optical density was read at a wavelength of 450 nm. The concentrations were assessed by interpolation from calibration curves prepared with standard samples provided by the manufacturer. As for the urinary analysis, we also measured urinary creatinine concentration (using enzymatic colorimetric method, kit Accent-200 Crea Enzymatic no 7–277, Cormay Diagnostics^®^, Warsaw, Poland, and biochemical analyzer Mindray BS-120, Shenzen, China) to provide objectivity of GSDMB urinary measurement.

### 2.2 Tissue samples

Skin samples were taken from the participants from the trunk using a 4 mm punch, after local anesthesia with 2% lignocaine. All the participants were advised to refrain from using any topical agents on their skin for a minimum of 1 month prior to the biopsy. Tissue samples were taken from 34 patients with psoriasis and 20 volunteers without skin diseases, matched for gender and age. For the patients, two biopsies were taken: one from the lesional skin, that is psoriatic plaque, and the other from unlesional, clinically healthy skin, that is about 2 cm from the psoriatic plaque. As for the controls, one skin sample was taken from clinically unaffected skin and performed while surgical removal of benign skin lesions. Subsequently, skin samples were fixed in a 10% buffered formalin solution. Following preservation, they were embedded in the paraffin blocks, and cut into 4 µm sections on silanized slides. It was followed by an overnight incubation at 60°C, deparaffinization, and rehydration of tissues. Slides were then incubated with 3% hydrogen peroxide solution (Peroxidase Block, Leica Novolink Polymer Detection System^®^, Illinois, United States) to block endogenous peroxidase and with protein block (Protein Block, Leica Novolink Polymer Detection System^®^, Illinois, United States) to avoid nonspecific antibody binding. Next, tissues were incubated with rabbit polyclonal anti-human GSDMB antibody (dilution 1:50, Sigma-Aldrich^®^, Massachusetts, United States, HPA023925) for 30 min at room temperature. Then, Post Primary Block and Novolink Polymer were used (Leica Novolink Polymer Detection System^®^, Illinois, United States). Protein expression was visualized with Novolink DAB solution and cell nuclei with hematoxylin (Leica Novolink Polymer Detection System^®^, Illinois, United States). Negative control of the staining was performed with rabbit immunoglobulin (Bond RTU Neg Rabbit, Leica, Illinois, United States).

**FIGURE 1:**
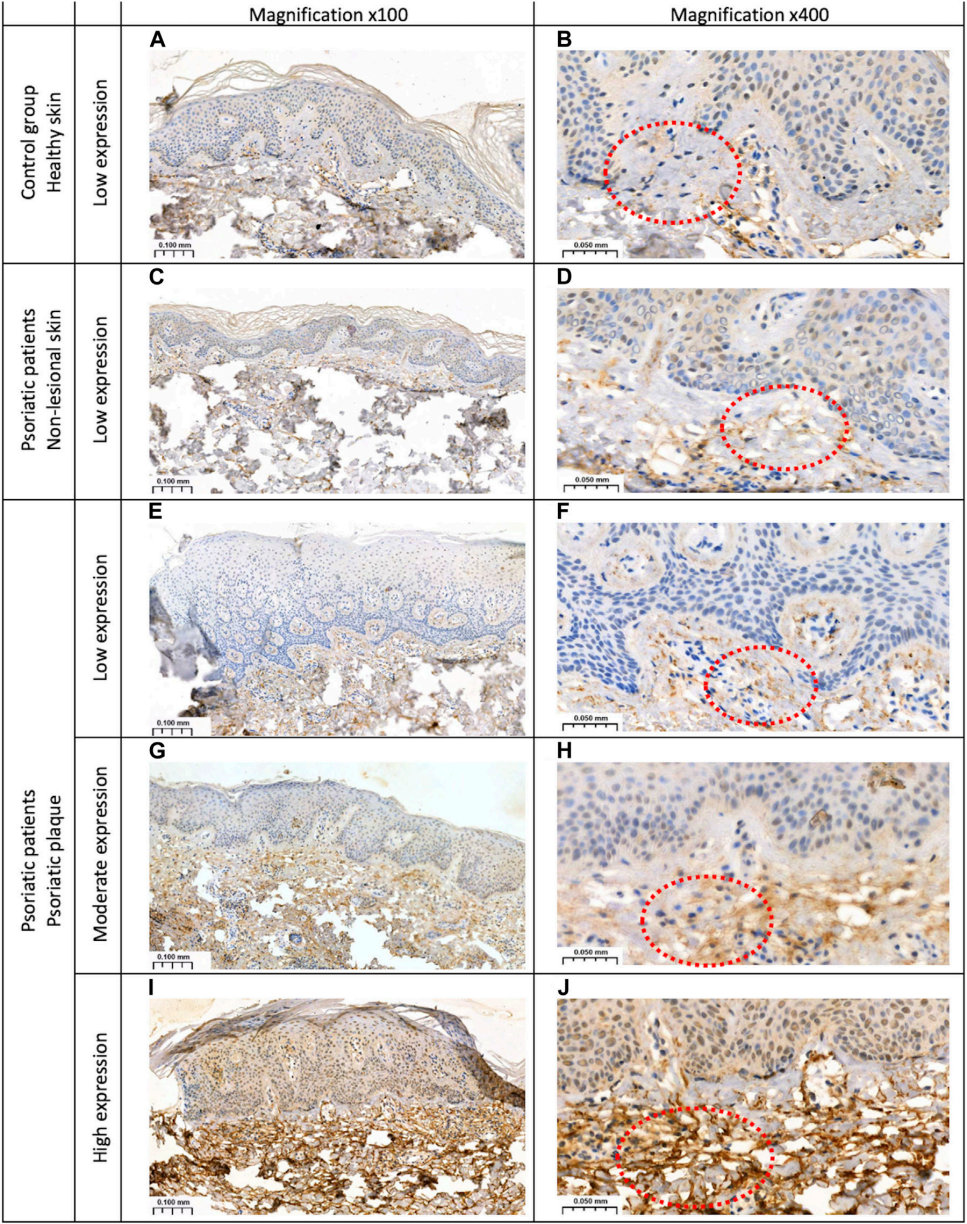
The expression of GSDMB: **(A, B)** low expression in the dermis of the healthy skin of controls; **(C, D)** low expression in the dermis of the non-lesional skin samples in patients; **(E, F)** low expression in the dermis within the psoriatic plaque; **(G, H)** moderate and **(I, J)** high expression in the dermis and epidermis within the psoriatic plaque. Magnifications ×100, x400.

Slides were scanned with Pannoramic 250 digital scanner (3DHISTECH, Hungary) and analyzed on SlideViewer software (3DHISTECH, Hungary). A semi-quantitative method was used to present the presence of GSDMB. The reaction was observed in the dermis and/or epidermis as follows: 1–low expression in the dermis in the elastic fibers (less than 10% stained area of dermis); 2–moderate (10%–30% stained area of dermis) or high (more than 50% stained area of dermis) expression in the dermis in the elastic fibers and the whole epidermis (nuclear-cytoplasmic reaction). Figure 1. Presents the expression of GSDMB.

### 2.3 Statistical analysis

All data were the subject of the analyses using the GraphPad 9.4 Prism. The normally distributed data were presented as mean ± SD and analyzed using a one-way analysis of variance (ANOVA). The non-Gaussian data were shown as median (full range) and analyzed using the non-parametric Kruskal–Wallis test. The differences between the psoriatic patients and the control group were compared by the Student’s t-test or nonparametric Mann–Whitney test. The relationship between two nominal variables was evaluated by the chi-square test of independence. The Spearman’s rank test was used to assess the correlations between the studied parameters. When the *p*-value is less than 0.05, differences were deemed to be statistically significant.

## 3 Results

The summary of the basic information about the participants is presented in Table 1.

**TABLE 1:** Basic characteristics of patients and controls.

Parameter	Controls (n = 30)	Psoriasis (n = 60)	*p*-value
Sex (M/F)	20/10	39/21	*p* > 0.9999
Age [years]	48 ± 2.45	50 ± 2.34	*p* = 0.6135
Height [cm]	1.75 (1.5–1.9)	1.71 (1.5–1.9)	*p* = 0.3929
Weight [kg]	78.40 ± 2.9	85.43 ± 2.5	*p* = 0.4137
BMI	25.85 ± 0.77	27.85 ± 0.64	*p* = 0.4137

There was no statistically significant difference between patients and volunteers from the control group in terms of age, gender or BMI (*p* = 0.6135, *p* > 0.9999, *p* = 0.4137, respectively).

### 3.1 Study on serum and urine

**FIGURE 2:**
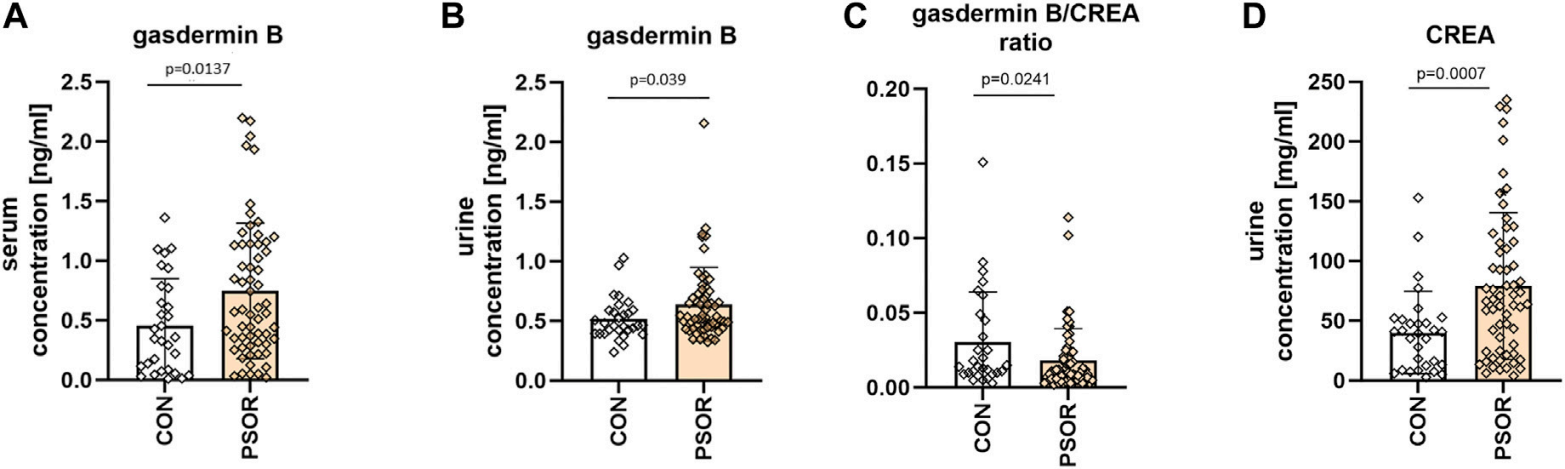
Serum GSDMB concentration **(A)**, absolute urinary GSDMB concentration **(B)** and urinary GSDMB/creatinine concentration ratio **(C)**, urinary creatinine concentration **(D)** in patients and controls. Serum and absolute urinary concentrations of GSDMB were significantly higher in psoriatic patients than in controls without skin diseases. The urinary GSDMB/creatinine concentration ratio was significantly lower in patients compared to controls. Box and error bar indicate the mean and standard deviation (SD).

The mean serum GSDMB concentration was 0.75 ± 0.57 ng/mL for patients and 0.45 ± 0.4 ng/mL for controls. The mean absolute urinary GSDMB concentration was 0.64 ± 0.31 ng/mL for patients and 0.52 ± 0.18 ng/mL for controls. The serum and absolute urinary concentrations of GSDMB were significantly higher in psoriatic patients than in controls without skin diseases (*p* = 0.0137, *p* = 0.039 respectively) (Figures 2A, B). The mean urinary GSDMB/creatinine concentration was 0.02 ± 0.02 for patients and 0.03 ± 0.034 for controls. The urinary GSDMB/creatinine concentration ratio was significantly lower in patients compared to controls (*p* = 0.0241) (Figure 2C). Additionally, in order to count the mentioned urinary GSDMB/creatinine ratio we also analyzed creatinine concentration in urine (Figure 2D).

**FIGURE 3:**
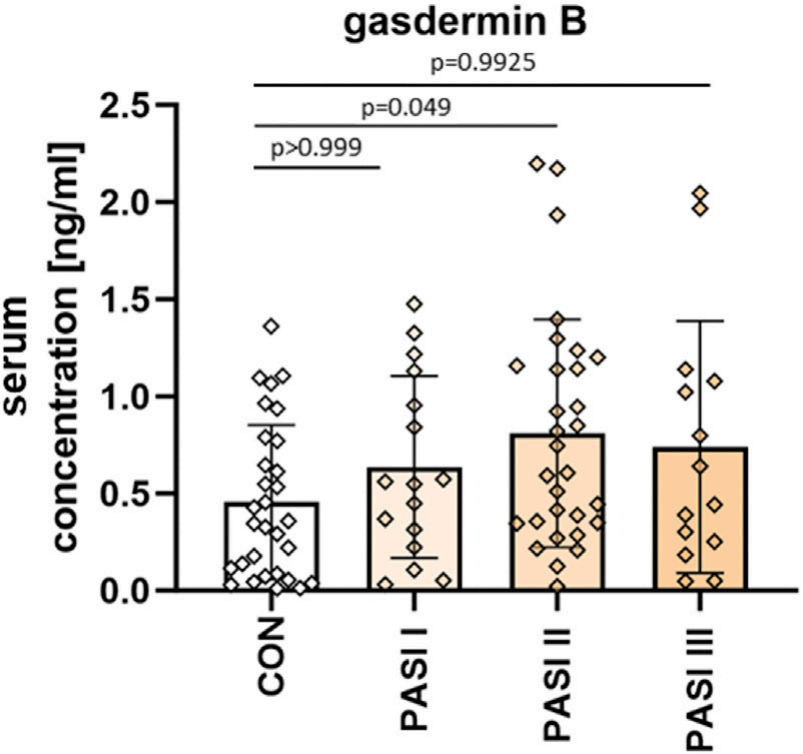
The division of serum GSDMB concentrations in patients depending on PASI compared to controls. No significant differences in GSDMB serum concentration between the three groups were found. Box and error bar indicate the mean and standard deviation (SD).

When considering the patients divided into three groups depending on psoriasis severity in PASI, the mean GSDMB serum concentrations were as follows: PASI I subgroup–0.64 ± 0.47 ng/mL, PASI II–0.81 ± 0.59 ng/mL, PASI III–0.74 ± 0.65 ng/mL. The GSDMB concentrations were higher in subjects with higher PASI but there was no significant difference between the three groups (*p* > 0.05) (Figure 3).

**FIGURE 4:**
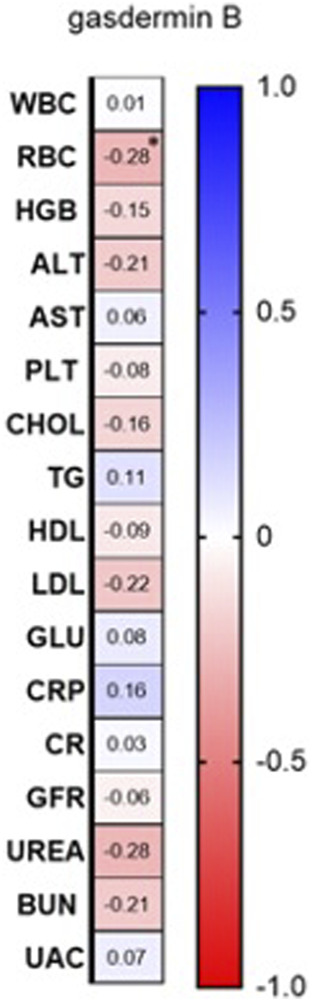
Correlations between serum GSDMB concentration and basic laboratory parameters. WBC, white blood cells [x10^3^/μl]; RBC, red blood cells [x10^6^/μl]; HGB, hemoglobin [g/dl]; ALT, alanine transaminase [U/l]; AST, asparagine transaminase [U/l]; PLT, platelets [x10^3^/μl]; Chol, total cholesterol [mg/dl]; TGs, triglycerides [mg/dl]; HDL, high-density lipoprotein [mg/dl]; LDL, low-density lipoprotein [mg/dl]; GLU, fasting glucose [mg/dl]; CRP, C-reactive protein [mg/l]; CR, creatinine [mg/dl]; GFR, glomerular filtration rate [ml/min]; BUN, blood urea nitrogen [mg/dl]; UAC, uric acid [mg/dl].

Among laboratory investigations, there was a negative correlation between GSDMB serum concentration and RBC (R = −0.28; *p* = 0.03) (Figure 4; Supplementary Figure S1).

**FIGURE 5:**
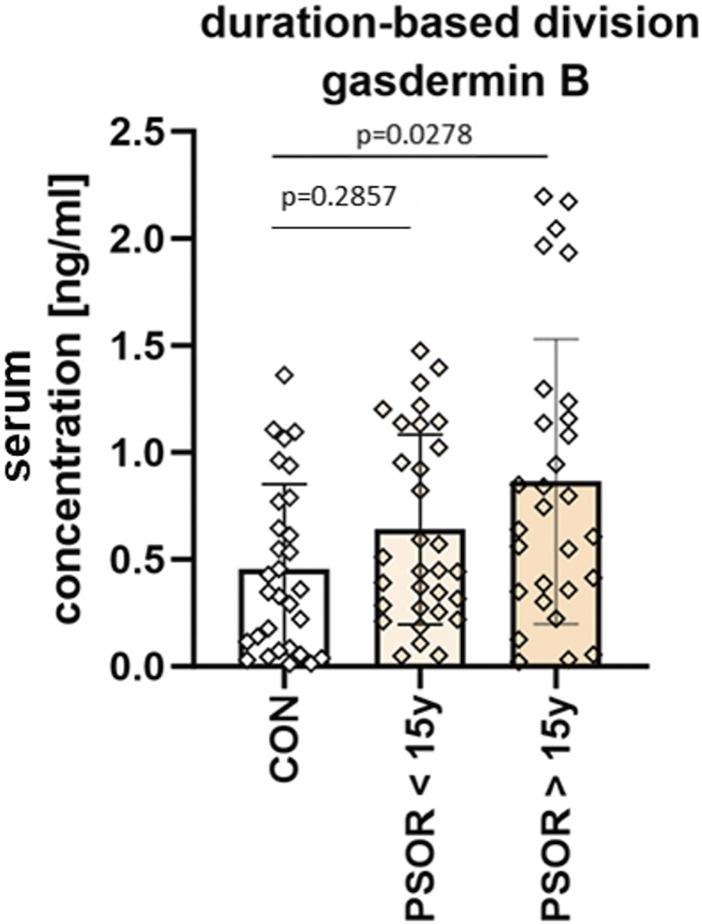
Division of patients based on disease duration. Serum concentration was significantly higher in patients with long-lasting psoriasis-more than 15 years, however insignificantly compared to the group with shorter duration. Box and error bar indicate the mean and standard deviation (SD).

The mean GSDMB serum concentration was 0.64 ± 0.44 ng/mL in subjects with short-lasting psoriasis-less than 15 years, and 0.86 ± 0.67 ng/mL in patients with long-lasting psoriasis-more than 15 years. GSDMB serum concentration was significantly higher in patients with long-lasting psoriasis (*p* = 0.0278), however insignificantly compared to the group with shorter duration (*p* > 0.05) (Figure 5).

Serum GSDMB correlated positively with the age of patients (R = 0.41; *p* = 0.001; Supplementary Figure S2, 3). GSDMB concentration was significantly higher in male than female patients (*p* = 0.0038). There was no correlation between GSDMB with BMI or PASI (*p* > 0.05).

### 3.2 Study on tissue

**FIGURE 6:**
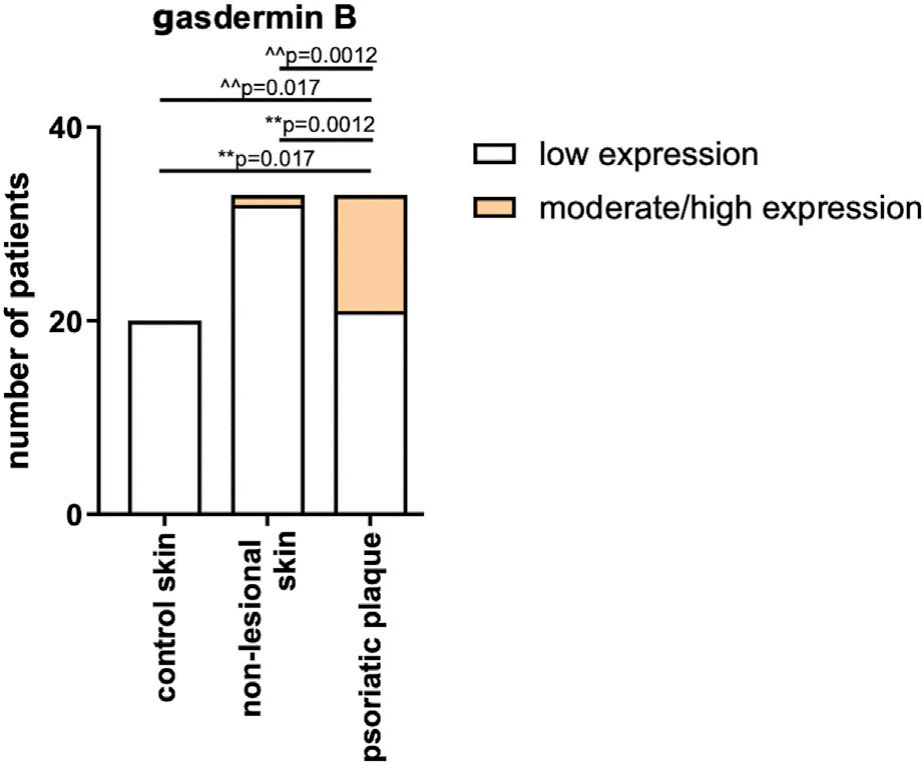
The expression of GSDMB in psoriatic plaque, non-lesional patients’ skin and healthy skin of controls. **vs. low expression, ^^ vs. moderate/high expression. Low expression in the dermis was observed significantly more frequently in the non-lesional skin than psoriatic plaques in patients, whereas there was not a single sample in the control group where moderate expression of GSDMB in the dermis and epidermis would be observed.

Skin samples were analyzed in 34 psoriatic patients, both in lesional and non-lesional skin, as well as in 20 subjects from the control group. In the control group, GSDMB expression was observed only in the dermis and it was low, similar in the non-lesional samples from the patients where GSDMB was expressed almost exclusively in the dermis, especially in the elastic fibers. Only in the psoriatic plaques, GSDMB was expressed both in the dermis and epidermis. The moderate/high expression of GSDMB in the dermis and epidermis was significantly more prevalent in psoriatic plaque compared to the non-lesional skin and healthy skin of controls (*p* = 0.0012, *p* = 0.017, respectively). Low expression in the dermis was observed significantly more frequently in the non-lesional skin than psoriatic plaques in patients (*p* = 0.0012), whereas there was not a single sample in the control group where moderate expression of GSDMB in the dermis and epidermis would be observed (Figure 6).

**FIGURE 7:**
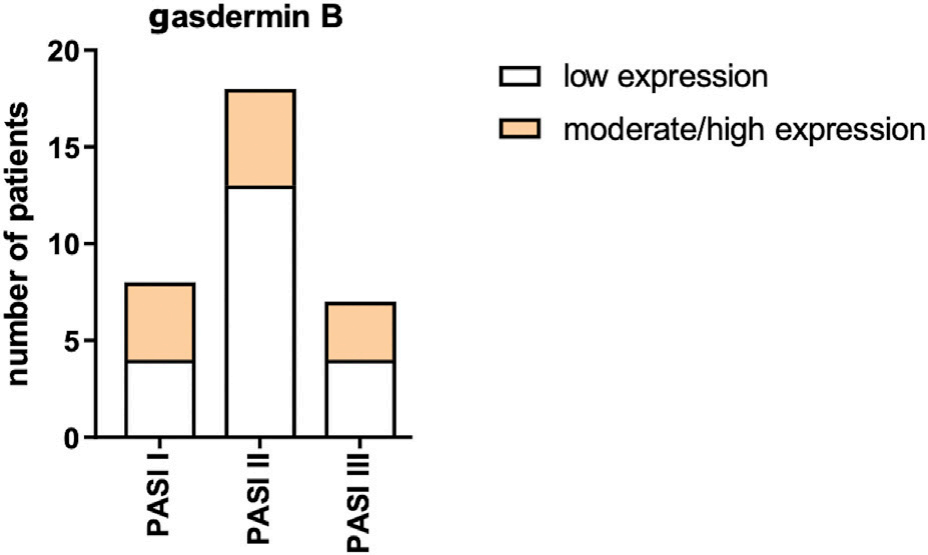
GSDMB expression in psoriatic plaque according to psoriasis severity in PASI. No significant differences in GSDMB expression between the subgroups were found.

After the division of patients according to psoriasis severity in PASI, there were no significant differences in GSDMB expression between the subgroups (*p* > 0.05) (Figure 7).

## 4 Discussion

GSDMB is expressed only in humans and seems slightly different from the rest of gasdermins (Zou et al., 2021), hence it requires a separate discussion. To the best of our knowledge, serum and urinary GSDMB concentrations, as well as its expression in immunohistochemistry, have never been investigated in psoriatics before.

The role of GSDMB still remains uncertain however, there are studies on its function in different disorders. Impaired GSDMB function may lead to uncontrolled inflammatory responses (Yin et al., 2023). Considering the association between psoriasis, IBD, and asthma, as well as documented engagement of GSDMB in IBD and asthma pathology (Chao et al., 2017; Das et al., 2017; Rana et al., 2022), we found it interesting to study this gasdermin in psoriatics. There is evidence of GSDMB upregulation in intestinal epithelial cells (IECs), especially colonocytes/crypt top colonocytes in patients with CD and UC, compared to controls without IBD (Rana et al., 2022). However, it also seems that GSDMB exerts a function other than enabling pyroptosis. Apparently, it promotes the migration of epithelial cells via the regulation of their adhesion (Rana et al., 2022). Furthermore, it has been shown that cells that lack GSDMB are characterized by lower proliferation (Rana et al., 2022). Our study revealed significantly more prominent expression of GSDMB in psoriatic plaques compared to uninvolved skin and controls, as well as higher serum GSDMB concentration which could indicate GSDMB role in psoriasis pathogenesis. Our idea is that, potentially, elevated levels of GSDMB indicate its stimulating effect on cell proliferation, whereas hyperproliferation of keratinocytes is a hallmark of psoriasis (Rendon and Schäkel, 2019). The epidermal turnover time in psoriasis is estimated to be about 4 days, whereas normally it is 28 days (Lowes et al., 2014).

Another disorder in which GSDMB has been investigated is asthma. Both asthma and psoriasis are related to IL17 signaling, chronic inflammation, impaired epithelium and barrier function (Busse, 2019; Östling et al., 2019). In psoriasis, IL17 is responsible for the hyperproliferation of the epidermis which we clinically perceive as scaly lesions (Furue et al., 2020). In asthma, IL17 is suspected to stimulate epithelial cells and fibroblasts to release neutrophil chemoattractants and granulocyte–macrophage colony-stimulating factor, resulting in neutrophilic infiltration in the lungs. Moreover, IL17A promotes the proliferation, migration, and contraction of smooth muscle cells. Interestingly, at the same time, an opposite, protective role of IL17 has been suggested, probably associated with the maintenance of epithelial integrity (Habib et al., 2022). Single nucleotide polymorphisms in GSDMB have been found to be associated with higher GSDMB expression which translates into asthma severity and a higher number of disease exacerbations (Li et al., 2021). The suspected role of GSDMB in asthma is based on apoptosis of epithelial cells regulation, hence cell proliferation, differentiation, moreover upregulation of the expression of airway remodeling genes, chemokines, and heat-shock proteins (Zhao et al., 2015; Li et al., 2021). Hence, there is another piece of evidence of the GSDMB engagement in epithelial cell turnover that could be a potential contribution to psoriasis pathology.

To our knowledge, there are only two other papers about GSDMB in psoriatic skin to date. In the first one, GSDMB gene expression was studied in a small number of psoriatic plaque samples and the highest expression was observed in the stratum basale of the psoriatic epidermis compared to other layers and it was higher than in controls (Zhang et al., 2022). In the second study, the expression of GSDMB in psoriatic epidermis was decreased compared to healthy skin (Ji et al., 2023). The authors of the latter study stated that decreased GSDMB in psoriasis leads to inhibition of cell proliferation and stimulates apoptosis (Ji et al., 2023). Taking into account that the lack of GSDMB had been linked to decreased cell proliferation in the past (Rana et al., 2022), psoriasis is known for decreased apoptosis (Shou et al., 2021), and those authors observed downregulation of GSDMB, these results do not correspond to psoriasis pathogenesis. Additionally, the results we obtained are the opposite. A more suitable explanation would be that GSDMB affects cell adhesion and controls cell proliferation, and considering we found it overexpressed in psoriasis, it is convergent with the increased proliferation rate of psoriatic epidermis. Moreover, our hypothesis matches what is observed in psoriasis histologically, namely, a widening of the intercellular spaces and a decreased expression of adherens junction proteins which lead to changes in adhesion between cells (Shutova et al., 2023). In case of IBD, the lack of GSDMB resulted in IECs hyperadhesiveness due to inhibition of cell motility (Rana et al., 2022). The opposite situation, confirming these findings, was observed in the study on breast cancer by Hergueta-Redondo et al. (Hergueta-Redondo et al., 2014). They found that overexpression of GSDMB in breast cancer cells is associated with increased cell motility and invasiveness so exhibits prometastatic influence (Hergueta-Redondo et al., 2014).

There is also evidence that IFNγ stimulates GSDMB expression (Zhou et al., 2020). At the same time, IFNγ is elevated in psoriasis and correlates with the disease activity (Grän et al., 2020). That could be another link between psoriasis and GSDMB, however, unfortunately, in this study, we did not find a correlation between PASI and serum GSDMB, as well as there were no differences between GSDMB expression in the psoriatic plaque depending on PASI, so it cannot be considered skin lesions severity marker.

As for another cytokine, that could provide a link between psoriasis and GSDMB, it has been demonstrated *in vitro* that overexpression of GSDMB in human bronchial epithelium upregulates some genes, including TGF-β1 (Das et al., 2017). This cytokine is also involved in psoriasis and increased plasma TGF-β is found in psoriatic patients (Flisiak et al., 2003).

In this study, we decided to examine GSDMB also in the serum and urine of all participants because they are collected in a less invasive way than a tissue sample and could be potentially used in clinical practice. Considering serum GSDMB and urinary GSDMB/creatinine concentration ratio were significantly different between psoriatic patients and controls, they could be further evaluated as potential psoriasis markers; in case of serum–an increased GSDMB, and in case of urine–a decreased GSDMB/creatinine concentration ratio. Obviously, it requires further validation but the application of urine in daily clinical practice would be non-invasive and widely acceptable by the patients. To the best of our knowledge, there is no other study in which urinary GSDMB would be analyzed.

GSDMB serum concentration was significantly higher in male patients and correlated with age, so perhaps its influence is more evident in older individuals.

It has been shown that GSDMB can also interact with the other gasdermin, namely, GSDMD. GSDMB is able to activate caspase four which further leads to cleavage of GSDMD (Chen et al., 2019). In our earlier experiment, which has been published (Nowowiejska et al., 2023b), we also studied the role of GSDMD in psoriasis and found it overexpressed in psoriatic plaque compared to healthy skin and suggested its role in psoriasis which seems convergent with what we observed now.

In this study, we found significant expression of GSDMB in the dermis, especially in elastic fibers. However, we have not found any other information about GSDMB role within the dermis and in relation to these particular fibers. The role of elastin in psoriasis has not been specified either. Therefore, the prominent presence of GSDMB in this skin layer remains to be elucidated.

The limitation of our study was a relatively low number of participants, especially in the group who donated tissue samples however, because of the invasiveness of this procedure, people are not always willing to take part in such investigations. Hence our idea is to use less invasive biological material, namely, serum or urine. Another limitation is the detection limit of the ELISA kit. An important issue that must be taken into account is that there are several isoforms of GSDMB uncovered and they may exert different functions (Sarrio et al., 2023). In this study, we examined total GSDMB concentration. The lack of precise data on the GSDMB dynamics in normal health conditions with regard to demographic parameters also impedes the interpretation of the obtained results.

In the future, we would also like to study the influence of classic antipsoriatic agents and biological drugs on GSDMB in psoriasis. We would also like to distinguish between cleaved and not cleaved forms of GSDMB.

## 5 Conclusion

GSDMB supposedly plays a different role in psoriasis than other gasdermins because its primary function seems to be not pyroptosis but regulation of cell migration. Considering our findings, namely, overexpression in psoriatic plaque compared to healthy skin and increased serum concentration compared to subjects without psoriasis, it is clear that GSDMB might be involved in psoriasis pathogenesis. Elevated serum GSDMB and decreased urinary GSDMB/creatinine concentration ratio could be potential psoriasis markers, associated particularly with cell migration. GSDMB could be considered in the future as an interesting potential therapeutic target.

## Data Availability

The raw data supporting the conclusion of this article will be made available by the authors, without undue reservation.
