# Correction: Gasdermin B (GSDMB) in psoriatic patients–a preliminary comprehensive study on human serum, urine and skin

**DOI:** 10.3389/fmolb.2026.1802468

**Published:** 2026-02-16

**Authors:** Julia Nowowiejska, Anna Baran, Anna Pryczynicz, Justyna Magdalena Hermanowicz, Beata Sieklucka, Dariusz Pawlak, Iwona Flisiak

**Affiliations:** 1 Department of Dermatology and Venereology, Medical University of Bialystok, Bialystok, Poland; 2 Department of General Pathomorphology, Medical University of Bialystok, Bialystok, Poland; 3 Department of Pharmacodynamics, Medical University of Bialystok, Bialystok, Poland

**Keywords:** gasdermin B, GSDMB, psoriasis, cell migration, keratinocytes, pyroptosis

In the published article, there was a mistake in the **Funding statement**. The **Funding Statement** for the National Science Center, Poland was displayed as “NCN/1/MI/22/001/1149.” The correct statement is “National Science Center, Poland, no **2022/06/X/NZ5/00356**.”

The original article has been updated.

