# Supplementary material for: Gasdermin B (GSDMB) in psoriatic patients–a preliminary comprehensive study on human serum, urine and skin
Source: Front Mol Biosci. 2024 Apr 17;11:1382069. doi: 10.3389/fmolb.2024.1382069 (PMC11061620; doi:10.3389/fmolb.2024.1382069)

SUPPLEMENTARY FILES

Figure 1. A diagram illustrating the correlation between serum GSDMB concentration and RBC in patients.

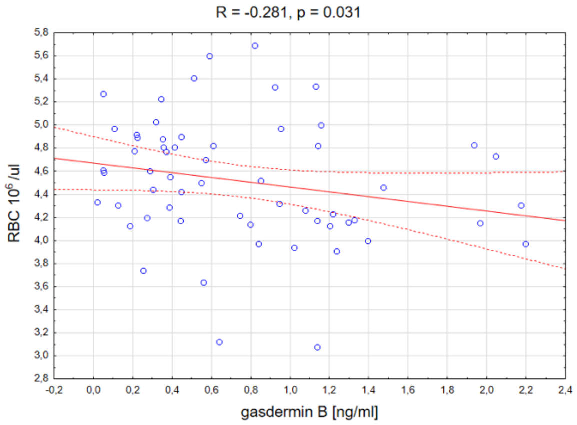

Figure 2. A scatter plot of age distributions of subjects in both groups. The circle and diamond indicate male and female.

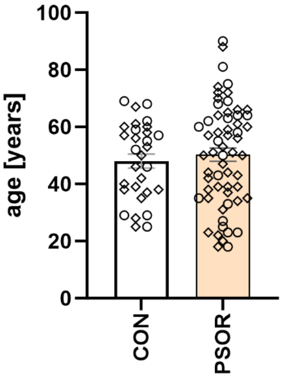

Figure 3. A diagram illustrating the correlation between serum GSDMB concentration and the age of patients.

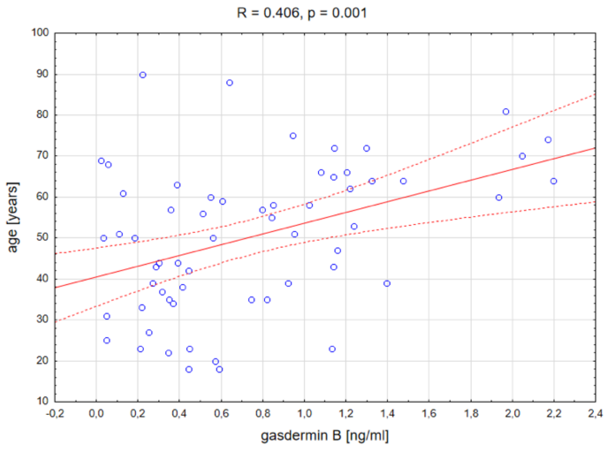

Supplement: Supplementary file 1 [file Image1.pdf]
